# Clinical characteristics distinguishing tramadol-using adolescents from other substance-using adolescents in an out-patient treatment setting

**DOI:** 10.1016/j.abrep.2020.100272

**Published:** 2020-03-31

**Authors:** A. Holmstedt, M.O. Olsson, A. Håkansson

**Affiliations:** a Lund University, Faculty of Medicine, Dept of Clinical Sciences Lund, Psychiatry, Lund, Sweden; b Malmö Addiction Center, Region Skåne, Malmö, Sweden; c Stockholm Center for Dependency Disorders, Region Stockholm, Centre for Psychiatric Research, Karolinska Institute, Stockholm, Sweden

## Abstract

**Background:**

Non-medical Prescription Opioid Use (NMPOU) has increased worldwide during the last decades, and specifically, tramadol misuse may represent a novel pattern of substance use among adolescents. The present study aims to analyze characteristics distinguishing tramadol-using adolescents from other substance-using adolescents seeking out-patient treatment.

**Methods:**

This is a cross-sectional study of treatment-seeking patients between 13 and 24 years of age in an out-patient facility for substance use problems in Malmö, Sweden. A total of 526 treatment-seeking adolescents at an out-patient treatment center were included. Data on substance use, treatment history and socio-demographic variables were extracted through a semi-structured interview method aimed specifically for adolescents with alcohol or drug problems (Ung-DOK). Lifetime tramadol users were compared to non-users, and also, primary tramadol users were compared to remaining subjects.

**Results:**

Thirty-one percent (n = 162) were tramadol users (lifetime prevalence). In logistic regression, the tramadol group showed a significantly increased risk of tobacco use, problematic lifetime cocaine, benzodiazepine and amphetamine use, and were more likely to report contacts with the judicial system, and less likely to report contacts with child or adult psychiatry, and more likely to have parents born outside the Scandinavian countries. In logistic regression, primary tramadol use was negatively associated with frequent cannabis use.

**Conclusions:**

Tramadol use appears to be a novel pattern among treatment-seeking adolescents. They showed a significantly increased risk of initiation of other illicit drugs and criminal behaviour, despite less contact with psychiatric care. More attention may be needed to this relatively novel pattern of opioid use.

## Highlights

• Tramadol is a common drug of misuse among substance-using adolescents.
• In young treatment-seeking patients, tramadol is more associated with polydrug use.
• Tramadol misuse is associated with sociodemographic differences and legal problems.

## 1 Background

During the last decades adverse consequences from non-medical prescription opioid use (NMPOU) have increased dramatically, such as in the United States, causing widespread public health problems (Cheng and DeBeck, 2017, Manchikanti et al., 2012, Saha et al., 2016). Researchers have described the occurrence of an American opioid epidemic. Among other factors, this may partly be due to the lowering of restrictions on the prescription of opioids for treatment of chronic non-cancer pain in the late 1990’s (Cheng and DeBeck, 2017, Manchikanti et al., 2012), and the promotion, in 1997–2002, of opioids by the pharmaceutical industry, leading to a 10-fold rise in prescriptions during this period (Executive Office of the President of the United States, 2017). The National Survey on Drug Use and Health (NSDUH) suggested that in 2016, 91.8 million US civilian, noninstitutionalized adults (34%) used prescription opioids; among whom 11.5 million (4%) misused them (Substance Abuse and Mental Health Services Administration, 2017). Researchers suggest, however, that an increasing awareness of NMPOU has made diversion and abuse of prescription opioids plateau and decrease in the last five years (Dart et al., 2015, Wilkerson et al., 2016). The numbers of heroin overdose-related deaths have escalated 4-fold in the US between 2010 and 2015 (Executive Office of the President of the United States, 2017), and although there was a slight decrease in heroin use in 2017 (Substance Abuse and Mental Health Services Administration, 2017), the transition from NMPOU to heroin abuse is a significant risk that should be considered, as available data indicates (Compton et al., 2016, Dart et al., 2015, Executive Office of the President of the United States, 2017, Jones, 2013, Wilkerson et al., 2016). While NMPOU is far from a US-only problem, direct comparisons between the US and European Union, for example, are difficult because of methodological differences in the drug abuse surveillance systems (Novak et al., 2016). While the most commonly used illicit opioid in Europe is heroin; however, a number of sources suggest that illicit use of synthetic opioids in Europe is increasing (European Monitoring Centre for Drugs and Drug Addiction Monitoring, 2018, Novak et al., 2016). In several European countries, non-heroin opioids represent the most common form of opioid use (Centre, 2018).

Tramadol is a central acting analgesic, and it is unique in that it acts through both opioid receptors and monoamine reuptake mechanisms. Tramadol inhibits serotonin and norepinephrine reuptake, enhancing inhibitory effects on pain transmission in the spinal cord. Due to tramadol’s modulation of norepinephrine and serotonin reuptake, the substance has specific risks which are atypical to standard opioid class pharmaceuticals. These adverse effects include tachycardia, hypertension, seizures and serotonergic syndrome (Bameri et al., 2018, Beakley et al., 2015, Hassamal et al., 2018, Taghaddosinejad et al., 2011, Yohei et al., 2015). The affinity of tramadol for the µ opioid receptor is low, approximately 10-fold less than that of codeine. The main metabolite of tramadol, O-desmethyltramadol (M1), has about 300-fold higher affinity to the µ opioid receptor than the parent compound. Conversion of tramadol to M1 is catalyzed by cytochrome P450 (CYP) 2D6. There is significant variability in the efficiency and amount of CYP2D6 enzymes among individuals. Phenotypic variations in CYP2D6 can be classified as poor metabolizers (PM) with little or no CYP2D6 function, Intermediate metabolizers (IM), extensive metabolizers (EM) and ultra-metabolizers (UM). UM and EM have approximately a 40% greater serum concentration of the active metabolite M1. Subsequently, UM and EM experience a stronger opioid response and a higher risk for addiction and overdose compared with PM and EM. PM have a 20% higher serum concentration of tramadol, and are at risk for adverse effects related to a hyperserotonergic state and seizures (Hassamal et al., 2018, Miotto et al., 2017).

Tramadol is recommended as a basic analgesic for the treatment of moderate to severe acute pain (Beakley et al., 2015, Frink et al., 1996, Grond and Sablotzki, 2004, Minami et al., 2015). In 2014, tramadol was added by the Drug Enforcement Administration (DEA) to category IV of scheduled drugs (Drug Enforcement Administration, Department of Justice, 2014). Researchers have found that tramadol enhances the reward system, thereby confirming its abuse potential (Babalonis et al., 2013, Smyj et al., 2013). Before the placement as a category IV scheduled drug tramadol was, by physicians, often considered to have a low potential for abuse and dependence with low adverse effects (Babalonis et al., 2013, Grond and Sablotzki, 2004). Mainly due to tramadol’s inhibitory effects on the serotonin and norepinephrine systems, research has shown potential use of tramadol as an antidepressant (Rougemont-Bücking et al., 2017, Tayal et al., 2008). Also, tramadol has an effect on the ejaculation latency in males with premature ejaculation. This is mainly due to tramadol’s inhibition of the serotonin transporter (SERT). The effect appears to be effective in the treatment of premature ejaculation (Esquivel-Franco et al., 2018, Martyn-St James et al., 2015); however, long-term tramadol abuse may be associated with poor sperm quality and hyperprolactinemia (Farag et al., 2018).

An increasing attention has been paid to adolescent use of tramadol, although markedly lower compared to the widespread use of cannabis; the prevalence of cannabis use is about five times that of other substances, and the most frequently used illicit drug among adolescents. In 15- to 16-year-old students from 35 European countries, 18% reported having used cannabis at least once. The use of illicit drugs other than cannabis (MDMA/ecstasy, amphetamine, cocaine, methamphetamine and hallucinogens) was far lower, with an overall lifetime prevalence of 5%. Lifetime use of sedatives or tranquilizers without a doctor’s prescription was reported by an average of 6% of students (Centre, 2018). In 2016, about 891,000 adolescents in the United States aged 12–17 misused opioids in the past year. This number corresponds to 3.6% of adolescents misusing opioids in the past year (Substance Abuse and Mental Health Services Administration, 2017). The knowledge of tramadol abuse among adolescents is limited. In the Middle East, studies confirm the widespread use of tramadol among adolescents (Bassiony et al., 2015, Bassiony et al., 2018), and in a study conducted in Sweden, Olsson et al. (Olsson, Öjehagen, Brådvik, Kronstrand, & Håkansson, 2017) concluded that tramadol misuse may represent a novel pattern of substance use among adolescents. While tramadol thereby appears to be a relatively novel pattern of NMPOU, including in young individuals, there is little research examining the characteristics of this increase and individuals reporting tramadol misuse. Therefore, the present study aimed to study characteristics of treatment-seeking adolescents reporting misuse of tramadol, compared to their counterparts seeking treatment for other substance use, and to study characteristics of primary tramadol users specifically, compared to the remaining treatment-seeking cohort.

## 2 Methods

### 2.1 Study design

The present study is an observational, non-interventional, cross-sectional study of tramadol use and its correlates within a cohort of adolescents and young adults seeking treatment at an out-patient facility for the treatment of substance use disorders in the young.

### 2.2 Clinical setting

Maria Malmö is an outpatient treatment center in Malmö, Sweden, aimed at adolescents and young adults with substance use disorders and problematic substance use. The upper age limit is 25 years, while there is no formal lower age limit. The treatment center is administered by both the social services and the health care services, and offers psychosocial and medical assessment, support and treatment, related to the abuse of drug and alcohol. The catchment area of the facility is the city of Malmö with approximately 340,000 inhabitants, the third largest city of Sweden. In Sweden, use and any handling of classified substances (addictive prescription drugs and illicit drugs) is illegal.

### 2.3 Measures

All adolescents included in the present study were assessed by staff at the center with the structured interview instrument Ung-DOK (‘Young-DOC’), which is the adolescent version of the DOC system. DOC (referred to in Swedish as DOK) stands for ‘documentation and evaluation regarding treatment of alcohol and drug abusers’ (Jenner & Segraeus, 2005). In the present study, data from this instrument were used in order to compare tramadol users to other individuals seeking treatment for a substance use disorder at the unit. The DOC instrument has an overall structure similar to that of the ASI (Addiction Severity Index), which is designed to measure the patient’s status in seven functional domains, and which characterizes and quantifies the severity of the multiple health and social problems (McLellan, Cacciola, Alterman, Rikoon, & Carise, 2006). Ung-DOK is a semi-structured interview method aimed specifically for adolescents with various forms of alcohol and drug problems (Dahlberg, Anderberg, & Wennerberg, 2017). The Ung-DOK interview contains questions within the following life domains: housing and financial support, occupation, tobacco use, alcohol use, drug use, treatment history, criminality, childhood, exposure to violence, family and relationships, physical health and mental health.

In the Ung-DOK questionnaire, primary drug is defined in the manual as the substance being the major problem to the individual at treatment start and/or the main reason why an individual seeks treatment, as judged by the interviewer when considering her/his own judgment and the opinion of the patient. Also, according to the interview manual, other substances are reported if they are judged from the patient assessment to constitute a problem. One exception here is that below 18 years of age, any alcohol use is always reported as a problem, whereas alcohol for majors is discussed and judged to be a problem or not depending on the interview. In the present paper, ‘use’ is defined as the reported problematic use of a substance according to the manual. The drugs specified in the Ung-DOK questionnaire are alcohol, cannabis, amphetamine, cocaine, ecstasy, LSD and other hallucinogenes, heroin, methadone, buprenorphine, GHB, spice (synthetic cannabinoids), solvents, benzodiazepines, other opioid and/or sedative drugs (also including analgesics without known addictive potential), and anabolic androgenic steroids. The category ‘other opioid and/or sedative drugs’ (reported by 14 percent of tramadol users and 10 percent of non-users, the difference being non-significant) was not further considered in the study. Also, the very rare non-opioid substances (GHB, and anabolic-androgenic steroids, both reported by one percent and non-significant across study groups), as well as LSD/hallucinogenes (non-significant across study groups, p = 0.636, reported by 7%) were not included for further analyses. The same goes for synthetic cannabinoids (reported by 17%, non-significant across study groups, p = 0.057), as this drug was reported virtually only during the first half of the study period, following a short-term rise in the use of this combination of drugs in the present community). Further, an ‘other’ category is available for other substances, i.e. a very wide ‘other’ group difficult to interpret, and not further considered in the present study, with the exception that the occurrence of tramadol from this category was coded specifically, and led to the grouping of tramadol users and non-users in the present study.

The frequency of drug use in Ung-DOK, for each problematic substance used, is reported in the categories no use, single occasion, one day a week or less, 2–3 days a week, 4–6 days a week, and daily. In the present study, the history of substance use during lifetime was used. Thus, the comparison of tramadol users and non-users refers to lifetime use vs non-use. In the secondary analysis, patients reporting tramadol as their primary drug were compared to all other individuals in the cohort. Other variables used in the present study include age, gender, whether parents are born outside Scandinavia, current (past three months) accommodation, past-three-month occupation, regular current tobacco smoking, lifetime history of having been convicted for crime, lifetime history of depression, anxiety/distress, difficulty controlling violent behavior, and current ongoing contact with adult or child and adolescent psychiatry.

### 2.4 Procedures

The Ung-DOK interviews were conducted by staff from Maria Malmö outpatient treatment center in Malmö, Sweden. The interviews were executed during 1st of January 2014 to the 31st of December 2017. All data from Ung-DOK were anonymous for the research group. The study was conducted only through retrospective analyses of Ung-DOK questionnaire data, and involved no repeated contact with the patients and therefore no informed consent procedure. The study was approved by the Regional Ethics Committee in Lund file number 2018/165.

### 2.5 Participants

Included patients were those seeking treatment at the Maria Malmö outpatient facility in Malmö, Sweden, and who had been interviewed with the Ung-DOK questionnaire, from 1st of January 2014 to the 31st of December 2017. During that period, a total of 635 Ung-DOK intake interviews were conducted. The completed Ung-DOK interviews were transferred to a database. A total of 107 interviews were identified as being the second or third interview of the same individual and therefore excluded, and two interviews were incomplete and therefore excluded from the study. This resulted in 526 Ung-DOK intake interviews being included in the study.

### 2.6 Statistics

The analyses compared characteristics of individuals seeking treatment with a history of problematic tramadol use, compared to the remaining patients. In a secondary analysis, those reporting tramadol to be their primary drug were compared to all patients reporting other primary drugs. Cross tabulation and multivariable logistic regression methods (the latter including variables which were significantly associated with tramadol use in the non-adjusted analyses) were used in the software SPSS (IBM SPSS statistics version 24). Pearson’s chi-squared test was applied for categorical variables for calculating differences between groups (Fisher’s exact test was used for the analyses of associations with the substances rarely reported). Results were considered significant if the p value was less than 0.05. For use of other substances than tramadol, any problem use (as defined by the Ung-DOK manual) during lifetime was used, however, as the problem use of cannabis was reported by nearly all participants, the variable addressing cannabis was set to describe frequent use (four times a week or more) vs all other clients. As the frequency variable is available for the past-three-month period, the variable distinguishing frequent from non-frequent cannabis use was studied for the past three months.

## 3 Results

A total of 388 respondents (74%) were men and 138 (26%) were women. The age varied between a minimum of 13 years to a maximum of 24 years. The median age was 17 years and mean age 18 years. A total of 431 (82%) had some form of occupation (school, work or work practice), whereas 93 (18%) had no form of occupation. Fourty-seven percent (n = 247) had both their parents born in Scandinavia (Sweden, Norway and Denmark) and 279 (53%) had one or both parents born outside the Scandinavian countries.

The most commonly reported primary drug was cannabis, reported by 425 subjects (81%), and 369 (70%) reported use of at least one drug other than the primary drug. Median age of onset of substance use was 15 years for cannabis (n = 507), 16 for tramadol (n = 162), 17 for amphetamine (n = 71), 17 for cocaine 17 (n = 133), 17 for benzodiazepines (n = 73), and 18 years of age for ecstasy (n = 105).

Among the 526 subjects, 162 (31%) stated any problematic tramadol use. Among them, past-three-month use was reported by 88 individuals (17%), and past-month use was reported by 58 individuals (11%). In binary comparison, lifetime tramadol use was significantly associated with age, occupation last three months, one or both parents born outside Scandinavia, ever being convicted, regular use of tobacco, a lifetime history of anxiety or distress, no ongoing contact with child or adult psychiatry, lifetime problematic use of cocaine, ecstasy, amphetamine, and benzodiazepines, as well as frequent cannabis use (on four days a week or more during the past three months, Table 1).

**Table 1:** Comparison of tramadol users and non-users. Chi-squared tests for categorical variables and *t*-test for age.

	Tramadol use (n = 162), n (%)	No tramadol use (n = 364)	Missing data	p-value
Age (Mean)	18.75	17.89	0	0.001
Gender				
Women	40 (25)	98 (27)		
Men	122 (75)	266 (73)	0	0.591
Occupation last three months	120 (74)	311 (86)	2	0.001
Own accommodation or with guardians last three months	139 (86)	315 (87)	1	0.763
Both parents born in Scandinavia	61 (38)	186 (51)	0	0.004
Ever convicted	67 (43)	70 (21)	39	<0.001
Regular use of tobacco	145 (90)	266 (74)	3	<0.001
Ever been depressed	123 (76)	244 (68)	8	0.062
Ever had anxiety or distress	129 (80)	248 (69)	8	0.012
Ever had difficulties controlling violent behavior	86 (53)	160 (45)	12	0.089
Ongoing contact with child or adult psychiatry	30 (19)	103 (28)	0	0.017
Problematic use, lifetime				
–alcohol	131 (81)	275 (77)	7	0.327
–cannabis	159 (98)	348 (96)	0	0.149
–cocaine	65 (40)	68 (19)	0	<0.001
–ecstasy	43 (27)	62 (17)	0	0.012
–amphetamine	38 (23)	33 (9)	0	<0.001
–benzodiazepines	41 (25)	32 (9)	0	<0.001
–heroin	6 (4)	9 (2)	0	0.411^1^
–buprenorphine	4 (2)	2 (1)	0	0.076^1^
–methadone	1 (1)	0 (0)	0	0.308^1^
–cannabis use 4 days a week or more	82 (51)	126 (35)	0	0.001

1 Fisher’s exact test.

In logistic regression, tramadol use remained significantly associated with having one or both parents born outside Scandinavia, ever being convicted, regular use of tobacco, absence of ongoing contact with child or adult psychiatry, and lifetime problematic use of cocaine, amphetamine, and benzodiazepines (Table 2).

**Table 2:** Associations with tramadol use in logistic regression.

Variables	OR	Lower	Upper	p-value
Gender	0.905	0.530	1.548	0.716
Age	0.958	0.868	1.058	0.397
Occupation last three months	1.566	0.857	2.862	0.145
Both parents born in Scandinavia	0.503	0.324	0.779	0.002
Ever convicted	1.687	1.022	2.022	0.041
Regular use of tobacco	2.646	1.445	4.844	0.002
Ever had anxiety or distress	1.234	0.729	2.089	0.433
Ongoing contact with child or adult psychiatry	0.527	0.303	0.915	0.023
Problematic cocaine use	2.037	1.172	3.540	0.012
Problematic ecstasy use	0.593	0.307	1.147	0.131
Problematic amphetamine use	2.498	1.247	5.003	0.010
Problematic benzodiazepines use	2.570	1.382	4.778	0.003
Problematic cannabis use 4 days a week or more	1.389	0.892	2.162	0.145

Six percent (n = 29) reported tramadol to be their primary drug. In binary comparison, tramadol as primary drug use was significant associated with problematic cocaine use and significantly and negatively associated with cannabis use on four days a week or more (Table 3). In logistic regression, reporting tramadol as the primary drug use remained significantly associated with problematic cocaine use, and significantly and negatively associated with reporting frequent cannabis use (four days a week or more, Table 4).

**Table 3:** Comparison of patients with primary drug tramadol use, and patients with other primary drugs. Chi-squared tests for categorical variables and *t*-test for age.

Variables	Tramadol primary drug (n = 29), n (%)	Tramadol not primary drug (n = 497), n (%)	Missing data	p
Problematic cocaine use	12 (41)	121 (24)	0	0.040
Problematic cannabis use 4 days a week or more	6 (21)	202 (41)	0	0.033

**Table 4:** Associations with having tramadol as primary drug, in logistic regression.

	OR	Lower	Upper	p-value
Problematic cocaine use	2.712	1.375	5.966	0.013
Problematic cannabis use 4 days a week or more	0.314	0.123	0.803	0.016

## 4 Discussion

The aim of this study was to examine which factors distinguish adolescents using tramadol from other substance-abusing adolescents in an outpatient treatment setting. The main findings are the significant rate of polydrug use associated with tramadol use, the association between tramadol use and a history of criminal conviction, and sociodemographic factors related to tramadol use, such as having one or both parents born outside Scandinavia. In addition, the tramadol use group was less likely to report an ongoing psychiatric treatment contact.

One significant finding was the higher rates of polysubstance use in the tramadol group, compared to other treatment-seeking clients. Adolescents reporting tramadol use showed a significantly increased risk of problematic use of other illicit drugs. Even when controlling for other measures of clinical severity, we demonstrated a higher likelihood of tramadol users reporting problematic use of cocaine, amphetamine and benzodiazepines. The result is in line with previous conclusions from Olsson et al. (2017) in the same setting. In contrast, however, in the present study, in the sub-analysis assessing factors associated with the primary use of tramadol (as the main drug), the association with problematic cocaine use remained, although the prevalence of frequent cannabis use decreases for this category. This may indicate that in the group defining their tramadol use to be their main type of drug use, the extensive use of cannabis – reported by nearly all subjects in the study – tends to be partially replaced by tramadol intake. Altogether, the higher rates of polysubstance use in the proportion of treatment-seeking clients who reported tramadol use, calls for increased attention in this patient group, with this relatively recent trend in tramadol misuse, to their risk of also co-using or having used other substances.

Polysubstance use, defined as using multiple substances within a specified period of time is an understudied characteristic of adolescents substance use and more research is needed. Young, Glover, and Havens (2012) found a positive association between illicit drug use and nonmedical use of prescription medications, and polydrug use is more common among young people receiving treatment as previous studies shows (Windle and Windle, 2012, Young et al., 2012). For tramadol specifically, Nazarzadeh, Bidel, and Carson (2014) studied the prevalence of tramadol misuse in a sample of Iranian adolescents and assessed the relationship between tramadol and other substance use. They found tramadol to be a factor related to adolescents’ alcohol, cannabis and ecstasy use, thereby a factor associated with a polysubstance use pattern.

The onset age of tramadol is 16 years and was preceded only by cannabis. This may be seen as a novel pattern of substance use, since tramadol only has been available in Scandinavia since the late 90’s and started to appear at the present outpatient clinic during the last decade. Polydrug use with adolescent onset increases the risk for lifelong drug use and psychiatric disorders (Brook et al., 2002, McKelvey et al., 2017). More longitudinal studies are needed to gain a better understanding of the association between tramadol use and transition into more prevalent polydrug use. Also, although the link between tramadol and use of other substances was one of the most important findings of the study, it cannot be excluded that the use of tramadol, as well as the use of other substances, are the result of a third factor not assessed here, or that being prone to try different substances would also increase the risk of trying tramadol as one substance among these. Thus, the direction of the association between tramadol use and other substance use in the present study cannot be established. However, the age of initiation of use was lower for tramadol than for most other substances, and in the consideration of this novel type of opioid use pattern, it may be of importance to note that its clinical picture is likely to involve a more extensive polysubstance pattern than in other young individuals seeking substance use disorder treatment.

### 4.1 Criminal convictions

One finding of the present paper was the association between tramadol use and ever being convicted for crime. Most researchers agree that crime and drug abuse are related. DeLisi, Angton, Behnken, and Kusow (2015) find that juvenile drug use is significantly associated with a criminal career, and Walters (2014) argues that youth who engage in comorbid delinquency and early onset of substance misuse may be at greater risk of subsequent criminal involvement and substance misuse, compared to youth who only engage in crime or only misuse substances. In the current study, even when controlling for age and gender, there was a clear association between tramadol use and a history of being convicted. From the Ung-DOK material, it is not possible to determine what type of crime the adolescents is convicted of, and all adolescents in the study receive treatment for different degrees of drug abuse and therefore, to the same extent, may have had a risk of becoming arrested for drug possession or drug-related offenses.

Tramadol’s unique properties in that it acts through opioid receptors and monoamine reuptake mechanisms could have an impact on the adolescent’s behavior. As the antidepressant mood elevation effect (Tayal et al., 2008) in combination with the modulating role of opioid systems on anxiety (Colasanti, Rabiner, Lingford-Hughes, & Nutt, 2011) could facilitate a more daring behavior, it cannot be excluded that these properties of tramadol may have implications for mechanisms leading to crime convictions as assessed in the present study. More research is needed to better understand the underlying factors that contribute to the association between tramadol use and crime convictions, and whether this is explained by characteristics of the substance itself or by shared risk factors in the population, or both.

### 4.2 Socioeconomic factors

Tobacco, alcohol, cannabis and polysubstance use are common behaviors among adolescents, particularly those experiencing socioeconomic disadvantage. Redonnet, Chollet, Fombonne, Bowes, and Melchior (2012) argue that adolescents with lower socioeconomic position generally initiated substance use at a younger age. Svendsen, Fredheim, Romundstad, Borchgrevink, and Skurtveit (2014) found that immigrant status was associated with less persistent opioid use. Håkansson, Schlyter, and Berglund (2009) state that opioid use is more common among non-Nordic immigrants. Having one or both parents born in another country could lead to problems for families’ integration into society, which in turn can lead to low socioeconomic status. Language difficulties also may cause problems regarding education. Unemployment is generally higher among immigrants, which also could affect the socioeconomic status. Andrabi, Khoddam, and Leventhal (2017) argue that teens with lower socioeconomic status may be more likely to live in communities were illicit substances are more accessible. More research is needed to gain a deeper understanding of the socioeconomic factors of tramadol use.

### 4.3 Lower rates of psychiatric treatment contacts

Polydrug users are at elevated risk of psychological distress (Kelly, Chan, Mason, & Williams, 2015). In line with the main findings we anticipated to find an increased psychiatric comorbidity in the tramadol use group. Contrary to our hypotheses, there was no significant association between tramadol use adolescents and other substance-using adolescents with respect to psychiatric symptoms. Additionally, the tramadol use group had less ongoing contact with psychiatric care. This is concurrent with Olsson et al. (2017) findings that tramadol use patients did not display more extensive comorbidity problems than other substance-abuse adolescents. The antidepressant properties of tramadol (Rougemont-Bücking et al., 2017, Tayal et al., 2008) and its effects on psychiatric symptoms may be a contributing factor and merits more research in order to further understand the underlying reasons for this. Eighty percent of the respondents stated cannabis as the primary drug. Cannabis use is often associated with major depression, mood disorders, anxiety and psychotic disorder (Di Forti et al., 2019, Gobbi et al., 2019, Gobbi et al., 2019). In the present study preliminary rates of psychiatric comorbidity in this group were high. Thus, it may be difficult to demonstrate significant differences between the groups.

## 5 Implications

Considering the global spread of opioids and the early age of onset for tramadol, and considering the present findings of tramadol becoming the second most prevalent drug after cannabis in young treatment-seeking patients, it is important to screen for tramadol use. If tramadol use is detected, screening should be continued as there is a strong significance for use of other illicit substances. This applies for all relevant authorities within healthcare, social services and the judiciary system. Thus, even though use of different substances in this group may not happen during the same episodes in life, as almost half of lifetime tramadol users did not report the use of that substance during the past three months, those reporting any tramadol use in their lifetime can still be seen as a group with a high risk of engaging in polysubstance use. In addition, it is of great importance for prescribing physicians to take the abuse potential of tramadol under consideration when prescribing tramadol.

## 6 Strengths and limitations

To our knowledge this is the first study that systematically examines the characteristics of tramadol users in an entire population of treatment-seeking adolescents and young adults. However, this study has limitations, including the fact that the study is based only on one city in Sweden; thus, generalizability to other setting cannot necessarily be assumed. Here, further studies in other geographical settings may be needed. Also, all measures were based on self-reports, such that misclassification and under-reporting of sensitive behaviors such as substance use can occur, a matter that should be kept in mind when interpreting the findings. Also, the study has an entirely cross-sectional design, and more prospective research is needed to examine the longitudinal associations of tramadol use.

## 7 Conclusions

Tramadol use is a novel pattern among treatment seeking adolescents in the present setting. The tramadol-using adolescents differ from others substance-abuse adolescents in that they showed a significant increased risk of also using other illicit drugs, and they are at greater extent in contact with the judicial system, but not with the psychiatric treatment system. More research is needed to understand the underlying mechanisms of adolescent tramadol use.

## Funding

The present study was funded by grants from the Region Skåne hospital system, and by ‘Finsam’ a public association for support to developmental projects within the hospital and the social services systems. The funding bodies had no role in the research ideas behind the study, nor in the data collection or in the interpretation of results or in the writing of the manuscript.

## Declaration of Competing Interest

The authors have no conflicts of interest to report related to the present paper.

Unrelated to the present project, Anders Håkansson holds a researcher position at Lund University which is sponsored by the state-owned Swedish gambling operator Svenska spel as a part of that body’s responsible gambling policy, and has research funding from the research councils of the state-owned gambling operator (Svenska spel) and the state-owned alcohol retail monopoly Systembolaget. In prior research, Anders Håkansson was the national sub-investigator of a multicenter pharmacoepidemiological study which was conducted by the US research institute Research Triangle Institute, which received an overall funding from Shire pharmaceuticals. Negotiations with the sponsoring body were not conducted on a sub-investigator national level and Anders Håkansson received no personal fees from the funding body. Currently, preparations are ongoing for a collaboration between Anders Håkansson’s research group and Kontigo care, a company providing technological follow-up tools for clinical research, and in a study on the clinical follow-up of gambling patients, Kontigo care is planned to provide follow-up devices free of charge, however without any other funding or any personal fees to the researchers.
